# Sucrose substitution in cake systems is not a piece of cake

**DOI:** 10.1038/s41538-023-00225-y

**Published:** 2023-09-27

**Authors:** Thibault Godefroidt, Isabella M. Riley, Nand Ooms, Geertrui M. Bosmans, Kristof Brijs, Jan A. Delcour

**Affiliations:** 1 Laboratory of Food Chemistry and Biochemistry and Leuven Food Science and Nutrition Research Centre (LFoRCe), KU Leuven, Leuven, Belgium; 2 Puratos NV, Groot-Bijgaarden, Belgium

**Keywords:** Polysaccharides, Sugar alcohols

## Abstract

Successful sucrose replacement in cake systems requires thorough understanding of its functionality. Time-domain ^1^H NMR showed that water in the viscous aqueous phase isolated from cake batter by ultracentrifugation [i.e. the batter liquor (BL)] exhibits low mobility by its low T_2_ relaxation time (T_2,D_ RT). This is due to its interactions with sucrose or sucrose replacers. The T_2,D_ RT itself is positively related with the effective volumetric hydrogen bond density of sucrose or sucrose replacers. Sucrose additionally co-determines the quantity and viscosity of cake BL and thereby how much air the batter contains at the end of mixing. Like sucrose, maltitol and oligofructose provide adequate volumes of BL with low water mobility and thus sufficient air in the batter, while the rather insoluble mannitol and inulin do not. Differential scanning calorimetry and rapid viscosity analysis revealed, however, that, in contrast to sucrose and maltitol, oligofructose fails to provide appropriate timings of starch gelatinisation and protein denaturation, resulting in poor cake texture. The shortcomings of mannitol and oligofructose in terms of respectively ensuring appropriate gas content in batter and biopolymer transitions during baking can be overcome by using mixtures thereof. This work shows that successful sucrose substitutes or substitute mixtures must provide sufficient BL with low water mobility and ensure appropriate timings of starch and protein biopolymer transitions during baking.

## Introduction

The global cake market size was valued at USD 42.94 billion in 2019 and is anticipated to grow at a compound annual growth rate of 3.3% from 2020 to 2027^1^. Cakes are sugar-rich foods which may be categorised as foam-, batter-, or chiffon-type cakes based on their formulation and production methods^2,3^. Foam-type (e.g. sponge) cakes are made from flour, sucrose, eggs, and leavening agent^2^. Batter- (or emulsion-) type cream and pound cakes additionally contain an oil or a fat source, respectively^3^. Chiffon-type cakes are a combination of the two cake types.

In response to dietary guidelines^4^, the food industry aims to reduce/replace sucrose in food systems. In soft drinks, this is comparatively easier because sugar can be replaced by high-intensity sweeteners^5^. In cake recipes, it is much more difficult to replace sucrose because of its diverse functionalities in their production, and because high-intensity sweeteners such as rebaudioside A^6^ and stevioside^7^ merely provide sweetness.

**Fig. 1:**
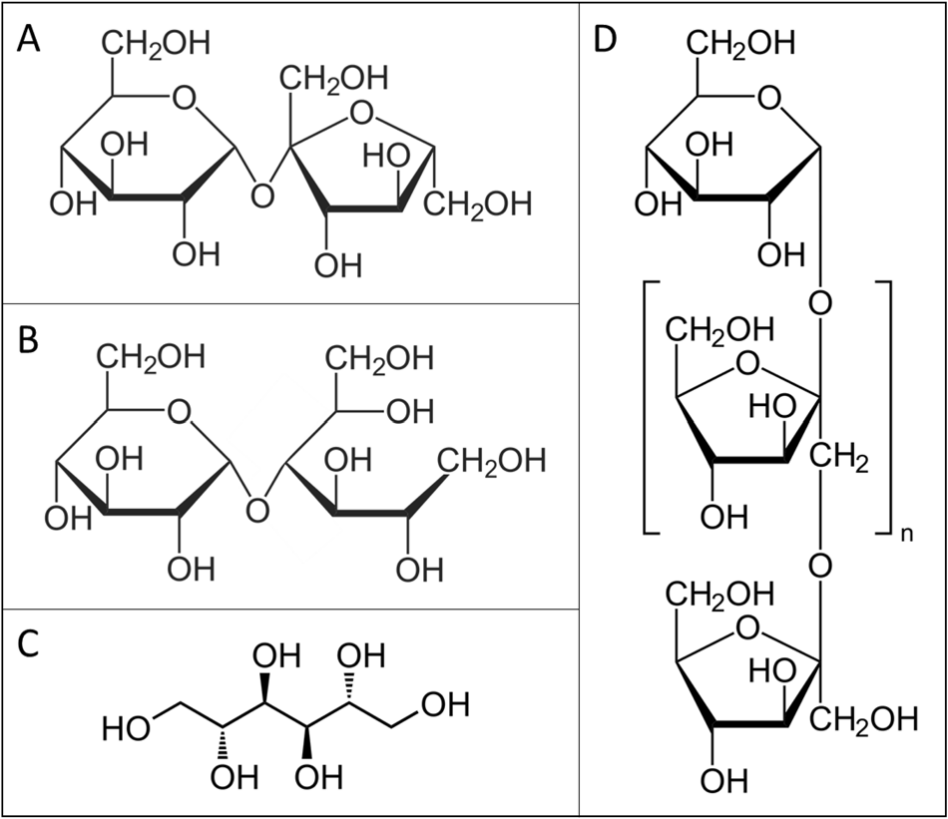
Structures of sucrose (substitutes). **A** Sucrose, **B** maltitol, **C** mannitol, and **D** oligofructose (*n* = 1–7) or inulin (*n* ≥ 8).

The cake-making process consists of mixing the ingredients into a batter and a baking phase of the batter. During mixing, air bubbles are incorporated in the batter, lowering batter density (BD)^2^. The air bubbles need to be sufficiently stable to ensure that the baked product has its desired volume^8^ and crumb texture^9^. For pound cake^7,10,11^ systems, high batter viscosity (η_B_) (measured with rotational^7^ or oscillating rod^10,11^ viscometers) has been associated with efficient air incorporation. Sucrose increases the viscosity of the aqueous phase of model foam systems, and thereby foam stability^8^. In sponge cakes, air is incorporated in the batter’s aqueous phase [further referred to as batter liquor (BL)]^12^. Against this background, we hypothesised that when using sucrose (substitutes), the quantity and quality of sponge cake BL, rather than the η_B_, are important determinants of air incorporation and batter stability during batter mixing. We thus examined whether BL fraction (i.e. the sum of water and sucrose divided by total recipe mass), viscosity (η_BL_)^13^, and proton (^1^H) mobility [analysed by time-domain (TD) NMR]^14^ are related to the incorporation of air in sponge cake batter and thus BD when using either less sucrose or the sucrose substitutes maltitol, mannitol, oligofructose, or inulin (Fig. 1). They all have lower calorie contents^15–17^ than sucrose, are bulking agents^15,16^ and, in case of mannitol and maltitol, also provide some sweetness^16,17^. Polyols such as maltitol may cause digestive issues when consumed in amounts exceeding 40 g/day^18^. Also, fibers such as oligofructose can induce browning during baking due to an increased amount of reducing oligosaccharides. To examine whether said potential negative effects can be reduced, mixtures of the previously stated sucrose substitutes are also tested.

While obtaining the right viscosity (η_B_ & η_BL_) may be important in cake making, it is not the sole parameter that determines cake quality^19^. During baking, the cake matrix sets due to wheat starch gelatinisation and protein denaturation^20–22^, the timings of which largely affect cake texture and quality^23^. Sucrose impacts the temperature at which starch gelatinises^24–29^ and protein denatures^30,31^. The assumed simultaneous occurrence of both phenomena^32^ transforms the liquid batter into a solid cake^33,34^. We here determined the effect of sucrose reduction/substitution on starch gelatinisation and protein denaturation using differential scanning calorimetry (DSC)^35^. Batter structure setting was studied by rapid viscosity analysis (RVA)^36^ and cake texture by texture profile analysis^37^.

We evaluated whether the findings for sponge cakes would also hold for batter-type cream and pound cakes. Based on our results, cake manufacturers are advised to select (mixtures of) sucrose substitutes which generate sufficient BL with low mobility and, in addition, ensure appropriate timings of starch gelatinisation and protein denaturation during baking.

## Results

### Sponge cake batter (liquor) properties

**Fig. 2:**
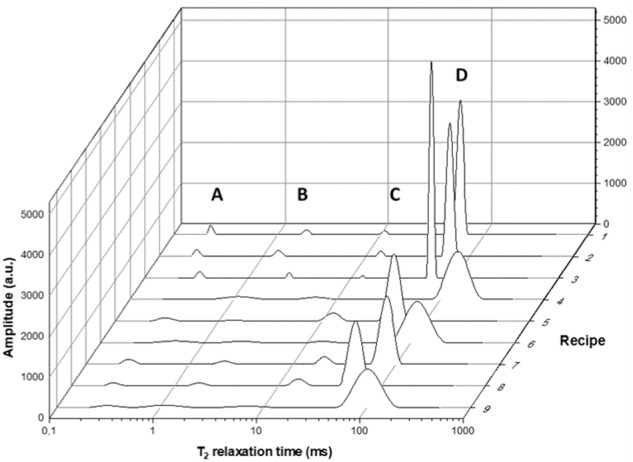
The proton distributions as determined by the Carr-Purcell-Meiboom-Gill (CPMG) pulse sequence for the different batter liquors (BLs). Recipe numbers are those of the different batters listed in Table 1. The proton populations are described by letters **A**–**D**, in order of increasing mobility. The amplitudes are provided in arbitrary units (au).

Sponge cake batters were made using different sucrose contents/substitutions (Table 1). The proton distributions of their recovered BLs (Fig. 2, Supplementary Data 1) revealed the effects of the different formulations at the molecular level. Proton populations in the Carr-Purcell-Meiboom-Gill (CPMG) pulse sequence were designated A to D in order of increasing mobility and assigned to dissolved compounds based on earlier work^38–41^. Populations A and B represent protons from minor constituents such as soluble starch (0–5% and 2–5% of total protons, respectively)^39,40^, while proton population C represents non-exchanging (CH) protons from dissolved sucrose^38^ or sucrose substitutes (2–11% of total protons). The most mobile fraction, proton population D, contained 82–93% of the protons. It mainly represents the hydroxyl group protons of water exchanging with those from sucrose^38,41^ or dissolved substitutes. Their T_2_ relaxation times (T_2,D_ RT) are a measure of hydroxyl proton mobility and evidently reflect the interaction of soluble components [mainly sucrose (substitutes)] with water^41^.

**Table 1:** Sugar (substitute) compositions of sponge cake batter recipes expressed on 100.0 g flour (14.0% moisture content) base, as well as rapid viscosity analysis (RVA) batter viscosity values^1^ (η_B_) of the batters at room temperature.

		Batter sugar (substitute) composition					
Recipe	Sugar (substitute)	Sucrose (g)	Maltitol (g)	Mannitol (g)	OF^2^ (g)	IN^3^ (g)	η_B_ (Pa.s)
1	*100% Sucrose*	87.0	0.0	0.0	0.0	0.0	7.7 (0.6)^a,b^
2	*70% Sucrose*	60.9	0.0	0.0	0.0	0.0	9.1 (1.1)^a,c^
3	*100% Maltitol*	0.0	87.0	0.0	0.0	0.0	8.0 (0.7)^a,b^
4	*100% Mannitol*	0.0	0.0	87.0	0.0	0.0	9.4 (0.2)^c^
5	*100% OF*	0.0	0.0	0.0	87.0	0.0	7.5 (0.8)^a,b^
6	*75/25 Mannitol/OF*	0.0	0.0	65.2	21.7	0.0	7.0 (0.8)^b^
7	*50/50 Mannitol/OF*	0.0	0.0	43.5	43.5	0.0	6.9 (1.0)^b^
8	*25/75 Mannitol/OF*	0.0	0.0	21.7	65.2	0.0	6.8 (0.8)^b^
9	*75/25 Mannitol/IN*	0.0	0.0	65.2	0.0	21.7	10.3 (1.2)^c^

^1^Values [*n* = 3; mean (standard deviations)] accompanied by different lowercase letters (a–c) indicate significant differences among samples (*p* < 0.05, Tukey’s test). ^2^OF: oligofructose. ^3^IN: inulin.

**Fig. 3:**
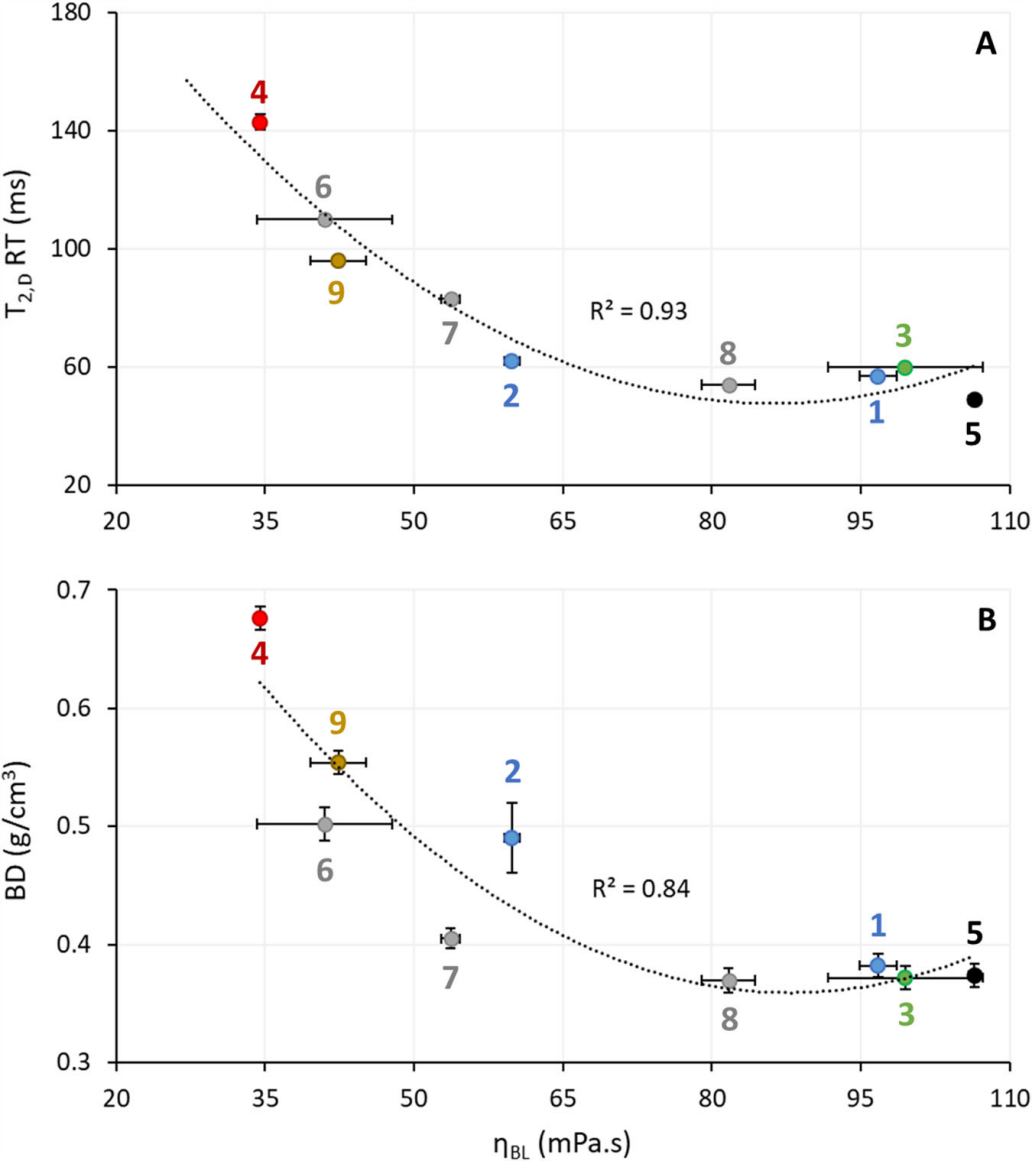
Plots of specific batter and batter liquor (BL) properties. Relation between (**A**) T_2,D_ relaxation time (RT) and BL viscosity (η_BL_) and (**B**) batter density (BD) and η_BL_. Recipe numbers are those of the different batters listed in Table 1. Error bars show the standard deviations from the means of three analytical replicates.

When reducing the sucrose content from 100% (control, *recipe 1*) to 70% (*recipe 2*), the batter contained less soluble material. The BL fraction indeed decreased from 62.1% to 56.8%. The mobility of the BL population D protons was slightly higher as evidenced by the higher T_2,D_ RT and lower η_BL_ (Fig. 3A) resulting from less strong water-sucrose interactions. Sucrose reduction also caused BD to increase (Table 2, Supplementary Data 2), but did not affect η_B_ (Table 1). This showed that the latter is a poor predictor of air incorporation during batter mixing.

**Table 2:** Batter densities, rapid viscosity analysis (RVA) temperatures^1^ of viscosity increase (T_η↑_) of sponge cake batters, differential scanning calorimetry (DSC) peak temperatures^1^ (T_p,batter_) of cream and pound cake batters, cake densities and textural parameters (springiness, cohesiveness, resilience, firmness) values^1^ from sponge cake (Table 1) and cream and pound cake (Table 4) recipes.

	Batter		Cake				
Recipe	Density (g/cm^3^)	T_η↑_ (sponge) / T_p,batter_ (cream and pound) (°C)	Density (g/cm^3^)	Crumb firmness (N*cm^3^/g)	Crumb springiness (%)	Crumb cohesiveness (%)	Crumb resilience (%)
*Sponge cake*							
1	0.38 (0.01) ^a^	91.4 (2.5)^a^	0.19 (0.01) ^a^	0.89 (0.04)^a^	85.9 (0.4)^a^	69.7 (0.7)^a^	21.9 (0.6)^a^
2	0.49 (0.03) ^b^	87.4 (0.6)^b^	0.25 (0.01) ^b^	2.90 (0.10)^b^	86.5 (0.5)^a^	66.6 (0.3)^b^	21.4 (0.2)^a^
3	0.37 (0.01) ^a^	88.7 (2.0) ^a,b^	0.19 (0.01) ^a^	0.82 (0.04)^c^	82.2 (0.2)^b^	66.3 (0.4)^b^	20.8 (0.5)^a^
4	0.68 (0.01) ^c^	90.7 (0.5)^a^	0.33 (0.01) ^c^	32.78 (1.68)^d^	43.7 (1.6)^c^	21.3 (0.3)^c^	5.0 (0.2)^b^
5	0.37 (0.01) ^a^	96.2 (0.6)^d^	0.19 (0.01) ^a,d^	0.84 (0.07)^a,c^	64.5 (3.5)^f^	55.7 (1.8)^e^	13.9 (1.6)^d^
6	0.50 (0.01) ^b^	86.1 (1.0)^b^	0.24 (0.01) ^b^	9.83 (1.40)^e^	40.2 (1.0)^d^	20.8 (0.6)^c^	4.0 (0.9)^b^
7	0.40 (0.01) ^d^	89.7 (2.8)^a,b^	0.21 (0.01) ^d^	1.02 (0.02)^f^	78.1 (0.4)^e^	60.6 (0.9)^d^	17.5 (0.1)^c^
8	0.37 (0.01) ^a^	94.7 (0.6)^c^	0.20 (0.01) ^a,d^	0.88 (0.11)^a,c^	76.7 (1.0)^e^	59.5 (0.6)^d^	17.0 (0.5)^c^
9	0.55 (0.01) ^e^	90.1 (2.5)^a,b^	0.29 (0.01) ^e^	16.86 (0.68)^g^	38.7 (0.5)^d^	19.9 (0.1)^f^	4.7 (0.2)^b^
*Cream cake*							
a	1.03 (0.01) ^a^	96.72 (0.59)^a^	0.49 (0.01) ^a^	10.27 (0.84)^a^	83.7 (0.6)^a^	61.4 (0.7)^a^	19.9 (0.5)^a^
b	1.00 (0.01) ^b^	97.49 (1.32)^a^	0.49 (0.01) ^a^	9.55 (0.88)^a^	81.7 (0.6)^b^	57.0 (0.6)^b^	17.3 (0.5)^b^
c	1.06 (0.00) ^c^	/	0.51 (0.01) ^b^	77.65 (7.63)^b^	47.7 (1.6)^c^	26.8 (1.0)^c^	7.2 (0.4)^c^
d	1.03 (0.01) ^a^	99.46 (0.63)^b^	0.48 (0.02) ^a^	11.38 (1.81)^a^	82.0 (0.7)^b^	58.3 (2.2)^b^	18.7 (1.0)^a,b^
*Pound cake*							
α	0.86 (0.01) ^a^	101.27 (1.43)^a^	0.45 (0.01) ^a,b^	10.95 (1.30)^a^	83.3 (0.8)^a^	59.7 (2.1)^a^	22.5 (1.3)^a^
β	0.86 (0.01) ^a^	101.53 (1.36)^a^	0.47 (0.01) ^a^	10.46 (1.24)^a^	80.2 (0.9)^b^	52.6 (1.8)^b^	18.3 (1.0)^b^
γ	0.86 (0.03) ^a,b^	/	0.40 (0.01) ^c^	44.16 (5.90)^b^	53.6 (1.8)^c^	30.2 (2.1)^c^	8.1 (0.6)^c^
δ	0.83 (0.01) ^b^	104.74 (1.96) ^b^	0.44 (0.01) ^b^	10.44 (1.28)^a^	82.1 (0.9)^a^	56.3 (2.4)^a^	20.7 (1.5)^a^

^1^Values [*n* = 3; mean (standard deviations)] accompanied by different lowercase letters (a–g) indicate significant differences among samples (*p* < 0.05, Tukey’s test); different cake types are separately compared.

Maltitol (*recipe 3*), like sucrose, completely dissolved in the BL (solubility ~200 g/100 ml), resulting in similar BL quantities and properties [i.e. T_2,D_, η_BL_, BD (Fig. 3, Supplementary Data 3), and η_B_ (Table 1)] to the control recipe. Mannitol (*recipe 4*) did not completely dissolve (solubility ~21.6 g/100 ml) resulting in less BL (estimated to be 43.2%) and lower η_BL_ (Fig. 3B). Accordingly, its T_2,D_ RT was substantially higher and the relaxation peak broader in shape than that of the control (Fig. 2), likely due to its poor solubility and the consequent heterogeneity in its interaction with water. The BD (Table 2) was significantly higher, and thus worse than noted for the control. When sucrose was replaced by oligofructose (*recipe 5*), DSC analyses (results not shown) indicated that it completely dissolved during batter mixing (as also observed earlier^15^), resulting in a BL fraction equal to that of *recipe 1* (62.1%) and a high η_BL_ (Fig. 3B). In this BL, the T_2,D_ RT was slightly lower than that of the control (Fig. 3A). The distribution of proton population D was broader (Fig. 2) because oligofructose is a heterogeneous mixture of different components. In these samples, for reasons not understood at present, the largest proportion of population C protons (about 10%) among all samples was noted. BD was low (Table 2) indicating high air incorporation in the batter.

Next, the impact of replacing mannitol (*recipe 4*) by oligofructose or inulin in increments of 25% (Table 1) was tested. Replacing 25% mannitol by oligofructose (*recipe 6*) led to improved air incorporation (reflected in the BD) and lower T_2,D_ RT (Fig. 3A). When 50% (*recipe 7*) or 75% oligofructose (*recipe 8*) were used with the corresponding mannitol complements, the mobility of water in the BL decreased even more, as reflected by their lower T_2,D_ RTs (Fig. 3A). The T_2,D_ RT of *recipe 8* and the control BL were very similar, as were their η_BL_ and BD (Table 2). When replacing 25% mannitol by inulin (*recipe 9*), DSC tests showed that, unlike oligofructose, it did not completely dissolve during batter mixing. While its BL and batter properties improved [lower T_2,D_ RT and higher η_BL_ (Fig. 3)] or remained comparable [η_B_ (Table 1)] to *recipe 4*, the BD remained inferior to *recipe 1* (Table 2). Inspection by the naked eye revealed that replacing more than 25% mannitol by inulin resulted in batters and cakes of exceedingly lower quality [i.e. BD, cake density (CD), and crumb firmness were high] and thus were not further considered.

This study shows that there are high correlations between η_BL_ and either T_2,D_ RT (R^2^ = 0.93) or BD (R^2^ = 0.84) (Fig. 3) and that there are no such correlations between either η_B_ and BD or T_2,D_ RT (results not shown).

### Biopolymer transitions during sponge cake baking

At high temperatures, a cake batter loses its fluidity and the cake structure starts setting as a result of starch gelatinisation and protein denaturation^33^.

During regular cake baking, both thermal events overlap^2,32^. The impact of sucrose (substitutes) on these endothermic transitions was examined, both separately and in batter, by DSC analysis. In the former case, the starch or egg white transitions were determined in excess water (*recipe 0*) and in media simulating the different BLs. In the latter case, the batters themselves were analysed. The resultant peak temperatures for wheat starch gelatinisation (T_p,starch_), egg white protein denaturation (T_p1,EW_ and T_p2,EW_), and the endothermic batter transition (T_p,batter_) are listed in Table 3 (Supplementary Data 4).

**Table 3:** Differential scanning calorimetry (DSC) peak temperatures^1^ of starch gelatinisation (T_p,starch_), egg white protein denaturation (T_p1,EW_ and T_p2,EW_) of different model systems containing either wheat starch or freeze-dried egg white in excess water (recipe 0) or excess media simulating recipe 1, 2, 3, 5, or 8 (Table 1) batter liquors (BLs), as well as the peak temperatures^1^ (T_p,batter_) observed during heating of the corresponding sponge cake batters.

	Model systems			Cake batters
Recipe	T_p,starch_ (°C)	T_p1,EW_ (°C)	T_p2,EW_ (°C)	T_p,batter_ (°C)
0	61.37 (0.14)^a^	66.61 (0.30)^a^	81.16 (0.04)^a^	83.58 (0.33)^a^
1	83.14 (0.46)^b^	75.46 (0.63)^b^	91.33 (1.21)^b,c^	99.33 (3.37)^b,c^
2	78.34 (0.58)^c^	73.59 (0.37)^c^	89.47 (0.40)^d^	91.50 (0.21)^d^
3	83.81 (0.25)^b^	76.44 (0.69)^b^	92.69 (0.54)^b^	98.60 (1.62)^b^
5	91.25 (0.59)^d^	75.22 (0.75) ^b^	91.59 (0.48)^b^	108.80 (2.90)^e^
8	85.96 (0.46)^e^	75.10 (0.62)^b^	90.67 (0.19)^c^	101.76 (0.92)^c^

^1^Values [*n* = 3; mean (standard deviations)] accompanied by different lowercase letters (a–e) indicate significant differences among samples (*p* < 0.05, Tukey’s test).

Compared to the water medium (*recipe 0*), biopolymer transitions occurred at increasingly higher temperatures in the sucrose-reduced (*recipe 2*) and sucrose-rich medium (*recipe 1*). No differences in the transition temperatures were noted between the sucrose-rich and maltitol-rich (*recipe 3*) media. The oligofructose-rich medium (*recipe 5*) caused egg white protein to denature at the same temperature as the sucrose-rich medium, while starch gelatinisation was delayed significantly (Table 3). This explains the significantly higher T_p,batter_ noted for *recipe 5* batter. The biopolymer transition temperatures of the 25% mannitol and 75% oligofructose batter (*recipe 8*) were most similar to those of the control batter (Table 3), indicating that a combination of the two substitutes can provide appropriate cake structure setting.

Unfortunately, the media simulating BLs containing more than 25% mannitol or inulin (*recipes 4, 6, 7, 9*; Table 1) could not be tested because their endothermic dissolution during heating interfered with the starch gelatinisation and egg protein denaturation endotherms.

### Sponge cake characteristics

**Fig. 4:**
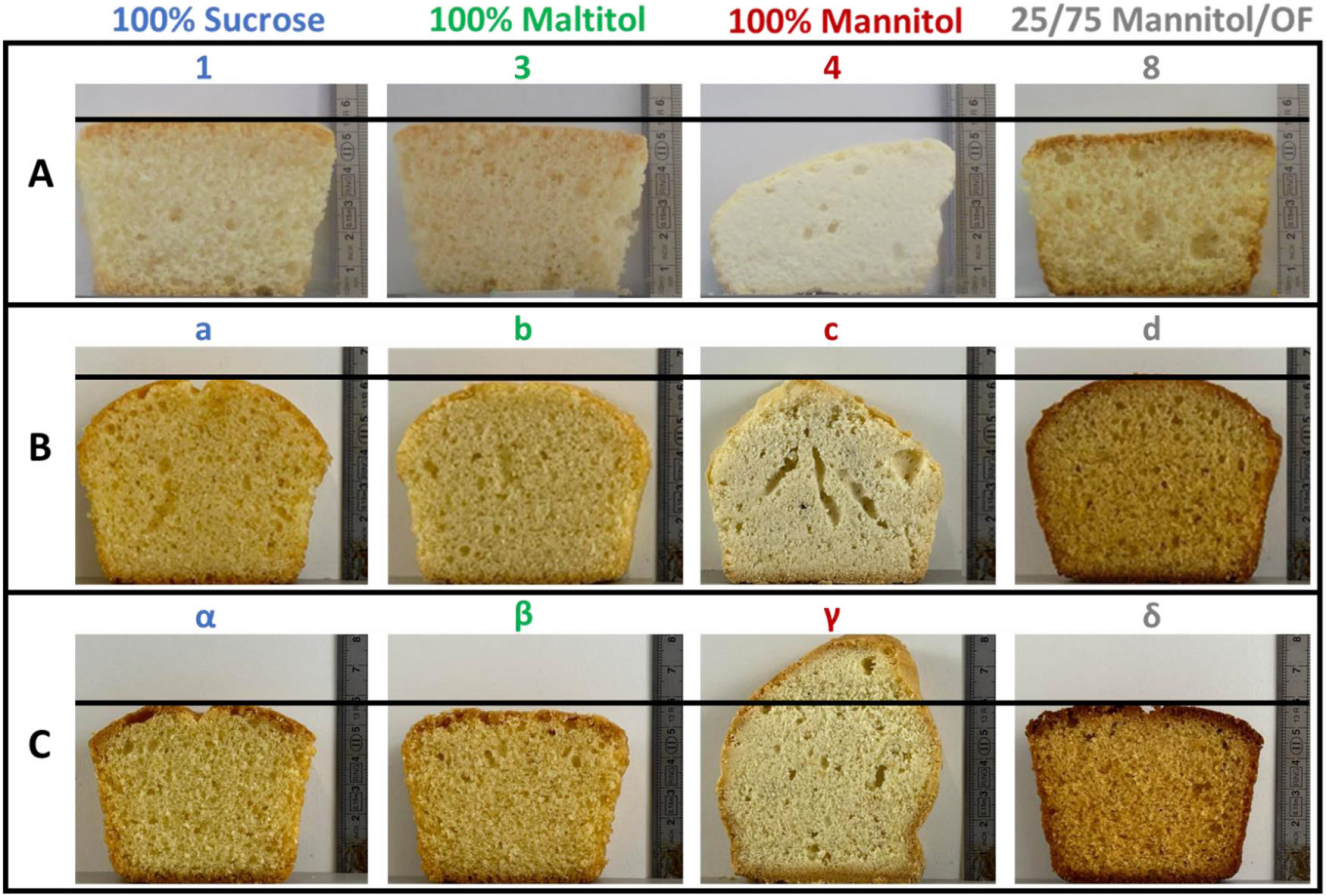
Photographs of intersection of different cakes. **A** Sponge, **B** cream, and **C** pound cakes prepared from selected recipes listed in Tables 1 and 4. A line is provided for each cake type at the max height of the control cake (100% sucrose) to compare their max height. OF oligofructose.

Cross-sections of sponge cakes from selected recipes are shown in Fig. 4A. The cakes made from control batter (*recipe 1*) had low CD and thus high volume with their texture properties taken as the benchmark for sponge cake (Table 2). When sucrose-content was reduced (*recipe 2*), the temperature of viscosity increase (T_η↑_, determined by RVA) was shown to decrease relative to the control batter and cakes were denser. Crumb cohesiveness and softness were negatively affected by sucrose reduction, while crumb springiness and resilience were not.

Cakes made with maltitol (*recipe 3*) displayed similar T_η↑_ and CD compared to control cakes (*recipe 1*, Table 2). Substitution with maltitol resulted in a similar cake crumb resilience and softness as determined for the control, and a lower crumb springiness and cohesiveness. Using mannitol (*recipe 4*), which minimally affects starch gelatinisation due to its low solubility^42^, resulted in poor cake quality as revealed by the significantly higher CD and poor cake crumb texture, even if the T_η↑_ was similar to that of the control (*recipe 1*). The use of 100% oligofructose (*recipe 5*) led to a higher T_η↑_ and thus delayed structure setting during baking (Table 2). While the CD was optimal, the crumb texture was suboptimal probably due to the significantly higher T_p,batter_^43^ than noted for the control and because starch gelatinisation and protein denaturation did not fully overlap (Table 3).

When mannitol was replaced by oligofructose in increments of 25% (*recipes 6* to *8*), CD and crumb softness were significantly better (Table 2). Other crumb characteristics also improved, however, even with 75% replacement of mannitol by oligofructose (*recipe 8*), they remained slightly inferior to those of the control cakes, in spite of their similar BL and batter properties (Fig. 3, Table 2).

Use of 25% inulin and 75% mannitol (*recipe 9*) resulted in a T_η↑_ comparable to that of the control and the 100% mannitol recipe (*recipe 4*). However, CD, crumb springiness, cohesiveness and resilience were not better than those of *recipe 4* cakes (Table 2). The slightly better gas cell incorporation during mixing led to average-wise thinner cell walls, and resulted in slightly softer crumb.

### Cream and pound cake making

To evaluate whether the present findings for foam-type cakes would also hold for batter-type cakes, cream (Fig. 4B) and pound (Fig. 4C) cakes were produced from recipes containing either sucrose, maltitol, a mixture of 25% mannitol and 75% oligofructose, or mannitol.

Replacing sucrose (*recipe a*) by maltitol (*recipe b*) in cream cake resulted in marginally better BD (Table 2). While the T_p,batter_ and CD of maltitol cream cakes did not differ from the control, their texture was inferior. Mannitol (*recipe c*) usage resulted in similar but less pronounced trends as observed for sponge cake (slightly worse BD and CD, significantly worse cake texture). Use of 25% mannitol and 75% oligofructose (*recipe d*) resulted in a BD and CD similar to those of the control, however, structure setting occurred slightly later.

In pound cake making, replacing sucrose (*recipe α*) by maltitol (*recipe β*) brought about similar BD and CD values (Table 2). While the biopolymer phase transitions of the batters were highly comparable, maltitol once again detrimentally affected cake texture. When sucrose was substituted by mannitol (*recipe γ*), BD did not differ. For reasons not understood at present, CD was better, however, as also noted for sponge and cream cakes, there was a significant negative effect on crumb texture. The use of 25% mannitol and 75% oligofructose (*recipe δ*) resulted in a slightly better BD and a similar CD as noted for the control. Structure setting was once again slightly delayed due to oligofructose use.

The observations for cream and pound cake thus aligned well with those for sponge cake.

## Discussion

**Fig. 5:**
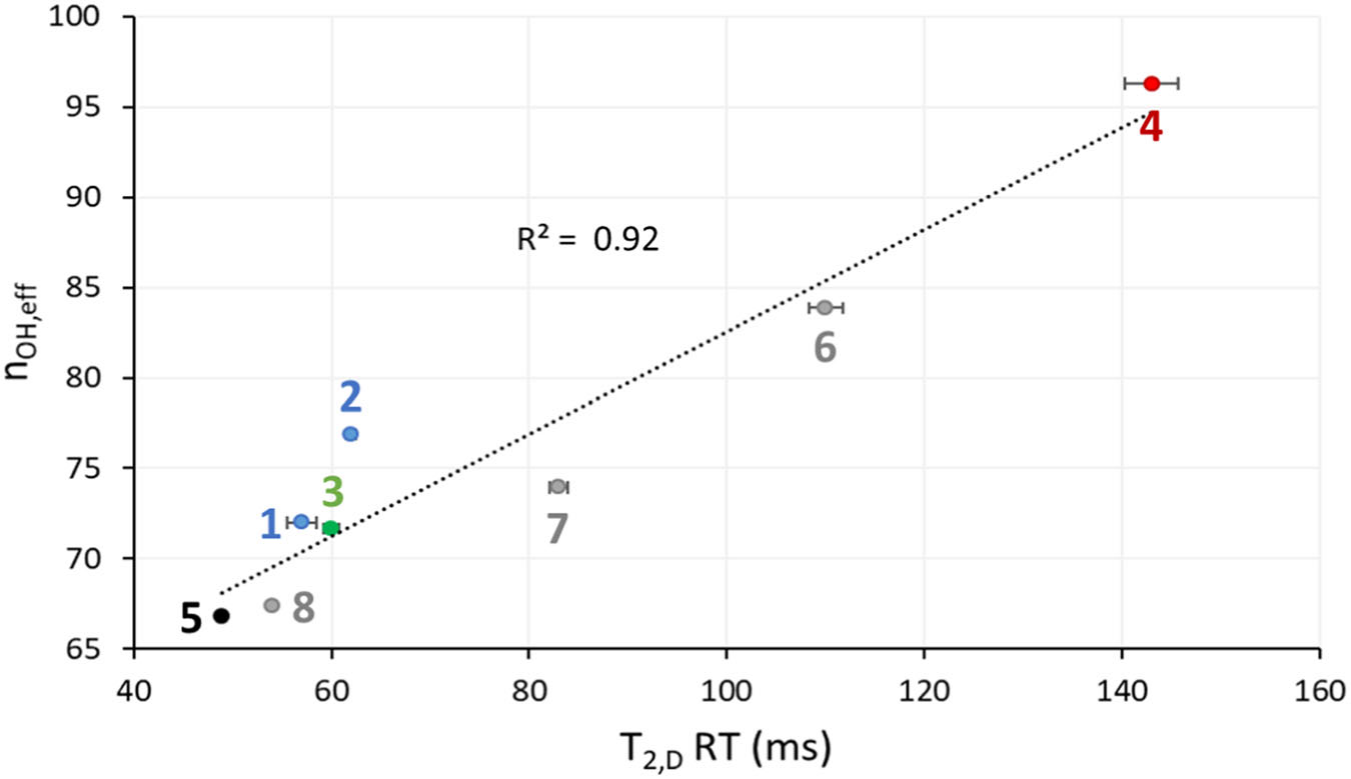
Plot of effective volumetric hydrogen bond density (n_OH,eff_)^44^*versus* T_2,D_ relaxation time (RT). Recipe numbers are those of the different batters listed in Table 1. Error bars show the standard deviations from the means of three analytical replicates of T_2,D_ RT.

Solubility of sucrose (substitutes) in the batter’s aqueous phase significantly affects the BL quantity, proton mobility in the BL, and η_BL_. Batters from recipes containing high amounts of sucrose (*recipe 1*) or soluble sucrose substitutes maltitol or oligofructose (*recipes 3, 5*, and *8*) have high BL quantities and evidently contain high amounts of air. At the same time, the mobility of their BL population D protons is low, η_BL_ is high (Fig. 3A), and the air is well retained in the foam/batter. Their resulting BDs are low. When the batters contain less sucrose (i.e. *recipe 2*), insoluble, or only partly soluble sucrose substitutes (i.e*. recipes 4, 6, 7, 9*), BL quantities and viscosities are lower, proton mobility in BL is higher, and batters are more dense. The T_2,D_ RT values are related to the volumetric density of hydrogen bonds in the aqueous phase (n_OH,eff_) (Fig. 5, Supplementary Data 5). As a result, n_OH,eff_ may be indirectly related to the η_BL_. The n_OH,eff_ parameter is important for explaining starch gelatinization and protein denaturation^44–47^ behaviour regarding the substitution of sucrose in cake systems. The high correlation between n_OH,eff_ and T_2,D_ RT (Fig. 5) underline how important it is to examine the intrinsic properties of the aqueous phase (i.e. η_BL_, T_2,D_ RT) when optimising sucrose substitution.

Importantly, the polynomial relation in Fig. 3B shows that a minimum η_BL_ of ±60–80 mPa.s is necessary to provide an appropriate BD. No such relation was found between η_B_ (Table 1) and BD. These results thus demonstrate that, to ensure sufficient gas cell incorporation and gas cell stability during batter mixing, BL quantity, η_BL_, and proton mobility – and not η_B_ – should be considered when replacing sucrose in sponge cake recipes.

In the control batter (*recipe 1*), starch gelatinisation and protein denaturation (Table 3) occur within a similar temperature range. When sucrose content is reduced (*recipe 2*), starch gelatinisation and protein denaturation occur at lower temperatures (Table 3) and batter leavening during baking is reduced, leading to cakes with higher CD and thicker gas cell walls^48^. Along with extensive amylose crystallisation^49^ after structure setting (due to more amylose leaching^50^), this causes cake crumb to be firmer (Table 2).

Sucrose and maltitol (*recipe 3*) have similar effects on the η_B_, starch gelatinisation^51^, and protein denaturation. The resultant cakes have comparable CDs and only a slightly different texture. Using mannitol (*recipe 4*) instead of sucrose leads to lower BL quantities, less gas cell incorporation during mixing, and detrimental effects on CD and cake crumb texture. Due to its poor solubility, mannitol does not delay starch gelatinisation and/or protein denaturation to a similar degree as sucrose. This explains its negative effect on cake quality. While using 100% oligofructose (*recipe 5*) has a positive effect on CD, starch gelatinisation is excessively delayed (Table 3)^43^, resulting in incomplete cake structure setting. The structural collapse and suboptimal crumb texture of these cakes likely results from starch gelatinisation and protein denaturation occurring at different moments during baking.

Step-wise replacement of mannitol (*recipe 4*) by oligofructose (i.e. *recipes 6, 7, 8*) positively affects CD and crumb texture (Table 2) due to the increased number of gas cells (with thinner gas cell walls) in the batter. When 75% of mannitol is replaced by oligofructose (*recipe 8*), the resultant BD is similar to that of the control (Table 2). However, the temperature of starch gelatinisation is slightly elevated compared to the control. The resultant cakes have an optimal CD and a similar crumb texture, except for a slightly reduced springiness and cohesiveness, the latter presumably due to the different timings of starch gelatinisation and protein denaturation. We conclude that the functionality of a mixture of oligofructose and mannitol in sponge cake making is similar to that of sucrose. Replacing 25% mannitol by inulin (*recipe 9*) marginally improves batter and cake quality (Table 2), and higher inulin concentrations result in significantly lower quality (data not shown).

When sucrose (*recipe A*, Table 4) is replaced by maltitol (*recipe B*) in cream cake, an almost identical effect as in sponge cake is observed regarding BD, CD, structure setting, and texture. In contrast to what is the case for sponge cake, mannitol has a limited effect on BD and CD in cream (*recipe C*) cake. In emulsion-type cake systems, gas cells are stabilised not solely in the aqueous phase, but also in the lipid phase^3^. Therefore, the effect of substitute solubility and η_BL_ on gas cell stability and BD is more limited, translating to mannitol-containing cakes having a similar CD as the control. Nonetheless, mannitol has a negative impact on cake texture, likely due to the altered phase transition temperatures of starch and protein compared to sucrose. Oligofructose (*recipe D*) positively impacts BD and CD, as also observed in sponge cake making.

**Table 4:** Sugar (substitute) composition of cream and pound cake batter recipes expressed on 100.0 g flour (14.0% moisture content) base.

Recipe	Sugar (substitute)	Sucrose (g)	Maltitol (g)	Mannitol (g)	OF^1^ (g)
Cream cake					
a	*100% Sucrose*	89.1	0.0	0.0	0.0
b	*100% Maltitol*	0.0	89.1	0.0	0.0
c	*100% Mannitol*	0.0	0.0	89.1	0.0
d	*25/75 Mannitol/OF*	0.0	0.0	22.3	66.8
Pound cake					
α	*100% Sucrose*	100.0	0.0	0.0	0.0
β	*100% Maltitol*	0.0	100.0	0.0	0.0
γ	*100% Mannitol*	0.0	0.0	100.0	0.0
δ	*25/75 Mannitol/OF*	0.0	0.0	25.0	75.0

^1^*OF* oligofructose.

When sucrose (*recipe α*, Table 4) is replaced by maltitol (*recipe β*), mannitol (*recipe γ*), or oligofructose (*recipe δ*) in pound cake (an emulsion-type cake), similar effects as those described for sponge cake are detected. Still, as noted above, mannitol has a different effect on CD in pound cake.

Thus, while the batter and BL properties of sponge cake (a foam-type cake) differ from those of emulsion-type cake systems in terms of gas cell stabilisation, the phenomena occurring during sponge cake baking and their impact on cake texture can be extended to cream and pound cake baking. This implies that both maltitol and mixtures of mannitol and oligofructose can be effective sucrose replacers in different cake systems.

The present findings regarding the intrinsic properties of the aqueous batter phase (i.e. η_BL_, T_2,D_ RT) and the biopolymer transitions and cake characteristics, along with the outcome of earlier research^47^, can serve as a basis for optimizing cake recipes, making sucrose replacement in these systems a bit more “a piece of cake”.

## Methods

### Materials

Wheat flour [moisture content, 14.0%; protein content, 11.6% (dry basis, N x 5.7)], maltitol, mannitol, leavening agent [sodium bicarbonate (NaHCO_3_) and sodium acid pyrophosphate (SAPP_15_)], and a monoacylglycerol and diacylglycerol based emulsifier were provided by Puratos (Groot-Bijgaarden, Belgium). SAPP_15_ releases 15% of the maximum amount of carbon dioxide (CO_2_) resulting from its reaction with NaHCO_3_ during the cake batter mixing phase^52^. Oligofructose [Orafti®P95, degree of polymerisation (DP) = 2–9] and inulin (Orafti®HP, DP ≥ 23) were provided by Beneo (Wijgmaal, Belgium). Wheat starch (moisture content 12.3%) was from Cargill (Sas van Gent, The Netherlands). Ultra-fine sugar, rapeseed oil, margarine, and hen eggs were obtained from a local supermarket. For some experiments, egg white was flash frozen with liquid nitrogen and freeze-dried. Freeze-dried egg white had a moisture content of 7.1%.

### Sponge cake batter making

The sugar (substitute) composition of the sponge cake batter are listed in Table 1. The recipes per 100.0 g flour further contained egg white (62.0 g), egg yolk (22.5 g), emulsifier (16.5 g), sodium bicarbonate (NaHCO_3_, 1.7 g), sodium acid pyrophosphate (SAPP_15_, 2.3 g), and water (35.2 g). The quantity of water per 100.0 g flour for the 70% sucrose sample (recipe 2) was 20.8 g so that all batters contained 35.5% water. Triplicate batters were made from 281.0 g wheat flour. Egg white and egg yolk were separated and weighed before use to allow accurate dosing. Wheat flour, sucrose or sucrose substitute, NaHCO_3_, SAPP_15_, and emulsifier were first manually blended in the mixing bowl of a Hobart N-50 5-Quart Mixer (Troy, OH, USA) until a homogenous dry mix was obtained. Water, egg white, and egg yolk were then manually folded into the dry component mix with a spatula to obtain a homogenous wet blend which was then mixed (300 s, 255 rpm, wire whip) to form aerated batter.

### Cream cake batter making

The sugar (substitute) composition of the cream cake batters are listed in Table 4. The recipes per 100.0 g flour further contained rapeseed oil (58.1 g), egg white (43.1 g), egg yolk (24.7 g), emulsifier (1.9 g), sodium bicarbonate (NaHCO_3_, 1.1 g), sodium acid pyrophosphate (SAPP_28_, 1.5 g), and water (43.7 g). Triplicate batters were made from 465.0 g wheat flour. Wheat flour, sucrose or sucrose substitute, NaHCO_3_, SAPP_15_, and emulsifier were first blended as above. Water, egg white and egg yolk, and rapeseed oil were then manually folded into the dry component mix with a spatula to obtain a homogenous wet blend. The blend was mixed (Hobart mixer) with a flat beater (120 s at 60 rpm; then 120 s at 124 rpm).

### Pound cake batter making

The sugar (substitute) composition of the pound cake batters are listed in Table 4. The recipes per 100.0 g flour further contained margarine (100.0 g), egg white (66.6 g), egg yolk (33.3 g), sodium bicarbonate (NaHCO_3_, 1.1 g), and sodium acid pyrophosphate (SAPP_28_, 1.5 g). Triplicate batters were made from 450.0 g wheat flour. First, the margarine and sucrose or sucrose substitute were blended (Hobart mixer, flat beater, 180 s at 255 rpm, i.e. the creaming step), after which the egg white and egg yolk were mixed in (30 s at 124 rpm). Finally, the wheat flour, NaHCO_3_, and SAPP_15_, were added and the blend was mixed (120 s at 124 rpm) to form batter.

### Determination of batter density

Batter densities (BDs) were calculated from the weights of 100 ml of two samples of each individual cake batter in tared plastic cylinders^53^. Averages of the six BD measurements are reported per batter.

### Isolation of sponge cake batter liquor and analysis of its viscosity

From each of the three individual sponge cake batters, six samples (20.0–30.0 g) in 38 mL thick polycarbonate tubes (Beckman Coulter, Brea, CA, USA) were centrifuged at 165,000 *g* (25 °C, 65 min) in a Beckman Coulter L7-65 Ultracentrifuge. From top to bottom, three phases were distinguished; a lipid, an aqueous, and a solid phase. The upper lipid phase was gently removed, and the aqueous phase was carefully decanted. The combined aqueous phase of each of the six samples per batter is further referred to as the BL.

The η_BL_ of each individual batter was determined in duplicate at 25 °C with a Brookfield Engineering (Middleboro, MA, USA) DV-III Ultra rheometer equipped with a cylindrical SC4-18 spindle (diameter 17.48 mm, length 31.72 mm). The cylindrical spindle was fully immersed in a BL aliquot (6.7 ml) in a SC4-13R sample chamber (diameter 19.05 mm) and then rotated at 10 rotations/min for 150 s. From 60 s onwards, the viscosity was recorded every 15 s for 90 s resulting in seven η_BL_ measurements per individual batter. The 14 readings of the η_BL_ of each tested batter were averaged.

### TD ^1^H NMR analysis of sponge cake BL

TD ^1^H NMR was used to study molecular mobility in the BLs. BL samples were accurately weighed (± 0.1 g) in NMR tubes (internal diameter 7.0 mm) and sealed to prevent moisture loss during analysis. The measurements were carried out with a Minispec mq 20 TD NMR spectrometer (Bruker, Rheinstetten, Germany) with 20 MHz operating resonance frequency for ^1^H (magnetic field strength of 0.47 T). The probe head temperature was 25 ± 1 °C. Because BL is an aqueous phase, relaxation curves were obtained using the CPMG pulse (90°, 180°) sequence. The lengths of the 90° and 180° pulses were 3.10 and 6.38 µs, respectively, with a pulse separation of 0.1 ms and 2,500 acquired data points. To obtain good signal-to-noise ratios, 32 scans were executed with a recycle delay of 4.0 s between consecutive scans. The signal decays were transformed to continuous distributions of T_2_ relaxation times with the CONTIN algorithm from Provencher^54^ (Bruker software) based on the inverse Laplace transformation.

The obtained proton populations were labelled in order of increasing mobility with alphabetic characters A to D and the assignment of proton populations was based on previous work from Luyts et al. (2013)^38^ and Pycarelle et al. (2020)^40^ on pound and sponge cake batter systems, respectively. The primary focus of the discussion was on CPMG proton population D (containing the exchanging protons of water and sucrose or sucrose substitutes) since it represented the largest fraction of protons in the proton distribution.

The weights of the BL samples were used for further calculations. For each proton population, the area and T_2_ relaxation time is reported, representing the relative amounts and mobility of protons in the corresponding population, respectively. The proton population areas are expressed in A.U. per g of BL. Analysis was performed on three subsamples from each sample, of which measurements were performed in triplicate.

### Effective volumetric hydrogen bond density calculation

Calculations of effective volumetric hydrogen bond density (n_OH,eff_) were done as in van der Sman & Mauer (2019)^44^. Molar mass (M), density (ρ), and hydrogen groups available for bonding (N_OH_) values were taken from van der Sman & Mauer (2019) and van der Sman et al (2022)^44,55^.

### Differential scanning calorimetry measurements

The effect of sucrose and its substitutes on gelatinisation of wheat starch and denaturation of egg white was studied in triplicate with differential scanning calorimetry (DSC) with a TA Instruments (New Castle, DE, USA) DSC Q2000. Wheat starch or freeze-dried egg white samples (2.5–3.0 mg) were accurately weighed in coated aluminium pans (Perkin Elmer, Waltham, MA, USA), after which water or water-containing media were added to obtain a wheat starch or freeze-dried egg white dry matter content of 25% (w_dm_/w_total_). Their composition was based on those of the BLs of *recipes 1, 2, 3, 5*, and *8* (*cfr*. Table 1) and thus contained 42.8% sucrose (w_sucrose_/w_total_), 36.3% sucrose, 42.8% maltitol, 42.8% oligofructose, and 42.8% (mannitol+oligofructose) respectively. The batters of said recipes were tested as well, by accurately weighing 6.5 – 8.5 mg of batter in coated aluminium pans. The pans were hermetically sealed, equilibrated at 0 °C, and then heated from 0 to 130 °C at 4 °C/min. A reference pan was heated simultaneously. The calibration was done with indium. Data were processed with TA Universal Analysis software. Unfortunately, the effect on starch gelatinisation, protein denaturation, and the batter could not be tested with DSC for samples containing more than 25% mannitol or inulin (*i.e. recipes 4, 6, 7*, and *9*), due to overlap of their dissolution peak with those of starch gelatinisation and egg white denaturation.

### Rapid viscosity analysis of sponge cake batter

Viscosity development during batter heating was analysed in duplicate with an RVA Super 4 (Perten, Hägersten, Sweden). The spindle was fully submerged in RVA cups filled with batter up to a height of 80 mm. Batter was then heated from 23 °C to 98 °C at 7.5 °C/min for 10 mins and further kept at that temperature for an additional 20 min. This heating profile was based on that in the centre of sponge cake batter when baked in a plate oven, as measured with a type T thermocouple (DataPaq, Cambridge, UK). The η_B_ was calculated from the current required to mix the batter with a paddle rotating at 75 rpm, as mentioned in Deleu et al. (2019)^56^. The temperature associated with a viscosity increase of more than 10% over the span of three consecutive 8 s intervals, i.e. T_η↑_, is here considered to be that at which structure setting started.

### Cake baking

Of each batter, 100.0 g (sponge cake) or 220.0 g (cream or pound cake) was added to three aluminium baking tins (lengthxwidthxheight, 170 × 75 x 50 mm) (Gents Bakkershuis, Ghent, Belgium). Batters were baked for 30 (sponge cake), 40 (cream cake), or 55 min (pound cake) in a plate oven (Hein Condilux, Hein, Strassen, Luxembourg) preheated to top and bottom temperatures of 180 °C and 160 °C (sponge and pound cake) or 180 °C and 180 °C (cream cake), respectively.

### Analysis of cakes

Baked cakes were cooled for 120 min at 23 °C and weighed prior to determining their volume with a VolScan Profiler (Stable Micro Systems, Godalming, UK). CDs of cakes were determined from their weights and volumes. Texture profile analysis^53,57^ was performed on the four most central 25 mm thick crumb slices of three cakes made from each batter. Cylindrical (30 mm diameter) crumb samples cut from the centre of these slices were compressed to 50% of their original height at 2 mm/s with an Instron (Norwood, MA, USA) 3342 Texture Analyser equipped with a cylindrical probe (diameter 75 mm) and a 50 N load cell, after which the samples were decompressed. The compression-decompression cycle was then repeated after a rest (3s). Crumb springiness, cohesiveness, resilience, and firmness were determined as in Godefroidt et al. (2021)^36^. The 12 measurements of each tested batter were averaged.

### Statistical analysis

The Tukey method (*P* < 0.05) was applied to detect differences in BD, CD, textural properties, and cake volume data using JMP Pro 16 (SAS Institute, Cary, NC, USA). The polynomial correlation was made using Microsoft Excel (Redmont, WA, USA).

### Reporting summary

Further information on research design is available in the Nature Research Reporting Summary linked to this article.

### Supplementary information

Supplementary Data 1

Supplementary Data 2

Supplementary Data 3

Supplementary Data 4

Supplementary Data 5

Reporting Summary Checklist

## Data Availability

All relevant data are included in the paper. All raw data are available from the authors upon request. Source data are provided with this paper.
